# Aspiration, stent retriever, or combined approach for basilar artery occlusion: a three-way comparative analysis

**DOI:** 10.1177/17562864251410787

**Published:** 2026-01-29

**Authors:** Muhammad Jaffar, Kazi Ahmed, Samir Abu-Rumeileh, Markus Otto, Lorenzo Barba, Thanh N. Nguyen, Mohamad Abdalkader, Piers Klein, Kyriakos Lobotesis, Mariarosaria Valente, Gian Luigi Gigli, Liqun Zhang, Matteo Foschi, Soma Banerjee, Giovanni Merlino, Robert Simister, Lucio D’Anna

**Affiliations:** Department of Stroke and Neuroscience, Charing Cross Hospital, Imperial College London NHS Healthcare Trust, London, UK; Department of Stroke and Neuroscience, Charing Cross Hospital, Imperial College London NHS Healthcare Trust, London, UK; Department of Neurology, Martin-Luther-University Halle-Wittenberg, Halle (Saale), Germany; Department of Neurology, Martin-Luther-University Halle-Wittenberg, Halle (Saale), Germany; Department of Neurology, Martin-Luther-University Halle-Wittenberg, Halle (Saale), Germany; Department of Neurology, Radiology, Boston Medical Center, Boston, Massachusetts, USA; Department of Neurology, Radiology, Boston Medical Center, Boston, Massachusetts, USA; Department of Neurology, Martin-Luther-University Halle-Wittenberg, Halle (Saale), Germany; Department of Neurology, Radiology, Boston Medical Center, Boston, Massachusetts, USA; Neuroradiology, Department of Imaging, Charing Cross Hospital, Imperial College London, NHS Healthcare Trust, London, UK; Clinical Neurology, Udine University Hospital and Department of Medicine (DAME), University of Udine, Udine, Italy; Clinical Neurology, Udine University Hospital and Department of Medicine (DAME), University of Udine, Udine, Italy; Department of Neuroscience, George’s University of London, Stroke, London, UK; Department of Biotechnological and Applied Clinical Sciences, University of L’Aquila, L’Aquila, Italy; Department of Neuroscience, Neurology Unit, S.Maria delle Croci Hospital, Ravenna, AUSL Romagna, Italy; Department of Stroke and Neuroscience, Charing Cross Hospital, Imperial College London NHS Healthcare Trust, London, UK; Department of Brain Sciences, Imperial College London, London, UK; Clinical Neurology, Udine University Hospital and Department of Medicine (DAME), University of Udine, Udine, Italy; SOSD Stroke Unit, Department Head, Neck, and Neurosciences, Udine University Hospital, Udine, Italy; SOSD Stroke Unit, Department Head, Neck, and Neurosciences, Udine University Hospital, Udine, Italy; Comprehensive Stroke Service, University College London Hospital, London, UK; Department of Stroke and Neuroscience, Charing Cross Hospital, Fulham Palace Rd, London W6 8RF, Imperial College London NHS Healthcare Trust, London, UK; Department of Brain Sciences, Imperial College London, London, UK

**Keywords:** aspiration, basilar artery occlusion, combined approach, stent retriever

## Abstract

**Background:**

Basilar artery occlusion (BAO) is a rare but devastating form of ischaemic stroke, with high rates of disability and mortality. While randomized trials have demonstrated the benefit of mechanical thrombectomy (MT) in BAO, the optimal first-line technique – aspiration, stent retriever, or a combined approach – remains undefined.

**Objectives:**

This multicentre study aimed to provide a three-way comparison of MT techniques in terms of efficacy, safety and subgroup-specific outcomes.

**Design:**

A retrospective observational study.

**Methods:**

We prospectively included 517 consecutive patients with acute isolated BAO treated with MT across seven comprehensive stroke centres between January 2019 and December 2023. Patients were grouped by first-line technique: aspiration (*n* = 200), stent retriever (*n* = 260), or combined approach (*n* = 57). The primary outcome was favourable functional outcome at 90 days (mRS 0–3). Inverse probability weighting (IPW) adjusted for baseline imbalances. Secondary outcomes included successful recanalization, excellent outcome (mRS 0–1), functional independence (mRS 0–2), mortality, symptomatic intracranial haemorrhage (sICH) and haemorrhagic transformation (HT). Predefined subgroup analyses were performed.

**Results:**

After adjustment, 90-day outcomes were similar across groups. Stent retrievers achieved higher recanalization rates (RR 1.86 vs aspiration, *p* < 0.001), while the combined technique was associated with less HT (RR 0.39 vs aspiration, *p* = 0.008). In patients ⩾80 years, stent-retriever use led to better outcomes than aspiration (39.2% vs 18%; *p* = 0.021). No other significant subgroup interactions were found.

**Conclusion:**

While overall functional outcomes were comparable, stent retrievers yielded superior recanalization and the combined technique reduced haemorrhagic complications. Technique selection may benefit from individualized, anatomy-driven decision-making. Randomized studies are warranted.

## Plain language summary

Comparing three techniques to remove blood clots in the basilar artery: a multicenter study of aspiration, stent retriever, and combined approaches

A stroke caused by a blockage in the basilar artery, a major blood vessel that supplies the brainstem, can be life-threatening and often leads to severe disability. Different techniques can be used to remove the clot and restore blood flow, but it is not yet clear which method works best. In this study, we analysed data from 517 patients treated for basilar artery blockage between 2019 and 2023 at seven stroke centres. Patients were treated using one of three approaches: aspiration, where suction is used to remove the clot; stent retriever, where a small device is used to pull the clot out; or a combined approach using both techniques. We assessed recovery 90 days after treatment, focusing on whether patients regained independence in their daily activities. Overall, the three techniques led to similar recovery rates, but there were some important differences. The stent retriever method achieved the highest success in reopening the blocked artery, while the combined approach had the lowest risk of bleeding in the brain. Among patients aged 80 years or older, treatment with a stent retriever resulted in better recovery compared to aspiration. These findings suggest that all three techniques are generally safe and effective, but the stent retriever may be preferred for older patients, and the combined approach may be considered when there is a higher risk of bleeding. Further studies, including randomized clinical trials, are needed to confirm these results and guide doctors in choosing the most appropriate treatment for each patient.

## Introduction

Basilar artery occlusion (BAO), accounting for approximately 1% to 4% of all ischaemic strokes, is a rare but clinically devastating subtype associated with high morbidity and mortality.^1–5^ Although meta-analyses of four randomized controlled trials have shown evidence in favour of mechanical thrombectomy (MT) for BAO,^6–11^ the optimal MT technique remains uncertain.^12^ Currently, three primary strategies are employed in MT: aspiration, stent retriever and combined techniques – first line. Trial designs to date have differed markedly: Basilar Artery Occlusion Chinese Endovascular (BAOCHE) relied almost exclusively on the Solitaire stent retriever, whereas ATTENTION (Endovascular Treatment for Acute Basilar-Artery Occlusion) allowed heterogeneous techniques, most commonly a combined approach incorporating both aspiration and stent retrieval. Therefore, no randomized data directly compared different MT techniques as first-line therapy in BAO. Previous observational studies and two meta-analyses generally demonstrated comparable functional outcomes for aspiration and stent-retriever techniques, although they have also linked stent-retriever use to higher rates of symptomatic intracranial haemorrhage.^12–16^ Thus, to date, available literature has been restricted to two-arm comparisons – aspiration versus stent retriever – without any direct three-arm evaluations that incorporate the combined technique. In this multicentre study, we conducted a trial emulation to perform a comprehensive three-arm comparative analysis of aspiration, stent retriever and combined thrombectomy techniques in patients with basilar artery occlusion. Focusing exclusively on BAO cases treated with endovascular therapy (EVT), we assessed clinical outcomes, safety profiles and procedural outcomes across all three approaches.

## Methods

### Database and population

This is an observational retrospective multicentre real-world study of prospectively collected data. The study followed the Strengthening the Reporting of Observational Studies in Epidemiology guidelines.^17^ We enrolled consecutive patients with acute ischaemic stroke due to isolated BAO undergoing MT from seven comprehensive stroke centres (Charing Cross Hospital, Imperial College Healthcare NHS Trust, London (UK); St George’s University of London, London (UK); University College London Hospitals, London, (UK); Martin-Luther-University Halle-Wittenberg, Halle (Saale), (Germany); Udine University Hospital, Udine, (Italy); Boston Medical Center, Boston, Massachusetts, (USA); AUSL Romagna, (Italy)) between January 2019 and December 2023. BAO was confirmed by baseline computed tomography angiography, magnetic resonance angiography, or digital subtraction angiography. Patients with concurrent occlusions involving the vertebral arteries, posterior cerebral arteries, or anterior circulation were excluded. In addition, cases with incomplete data on the thrombectomy approach were excluded from the analysis. The choice of thrombectomy technique was left to the discretion of the treating neuro-interventionalist. Peri-procedural intra-arterial medications included the administration of alteplase and nimodipine as indicated. Clinical decisions regarding treatment with MT and intravenous thrombolysis (IVT) were made in accordance with current national and international guidelines. Alteplase was used as the thrombolytic agent; tenecteplase was not administered at any participating centre. Clinical and radiological assessments were performed by local neurologists and neuro-interventionalists at each site. The modified Rankin Scale (mRS) score at 90 days was determined either through an in-person follow-up visit or via a structured telephone interview. All data were reviewed for plausibility, integrity and completeness using a standardized protocol. In cases of data inconsistency, queries were issued to the respective centres for clarification. Patients without available 90-day mRS (*n* = 30) were excluded from outcome analyses. Given the low proportion of missing follow-up data (5.3%) and the absence of clinically relevant differences in baseline characteristics between patients with and without follow-up, we performed a complete-case analysis without imputation.

### Data collection

Baseline demographic and clinical variables were systematically collected, including age, sex and pre-stroke modified Rankin Scale (mRS) score. Vascular risk factors comprised hypertension (defined as blood pressure ⩾140/90 mmHg on at least two separate occasions or current antihypertensive therapy), diabetes mellitus (history or fasting glucose ⩾126 mg/dL, postprandial glucose ⩾200 mg/dL, HbA1c ⩾6.5%, or antidiabetic treatment), hypercholesterolemia (total cholesterol ⩾200 mg/dL, triglycerides ⩾140 mg/dL, or use of lipid-lowering therapy), coronary artery disease, previous ischaemic stroke or transient ischaemic attack, heart failure (defined per Left ventricular ejection fraction- LVEF thresholds),^18^ smoking status (current or former), alcohol abuse^19^ and known or newly detected atrial fibrillation (atrial fibrillation detected after transient ischaemic attack or stroke or AFDAS). Pre-stroke antithrombotic use (antiplatelets and oral anticoagulants) was also recorded. Stroke severity at admission was assessed using the NIHSS and imaging findings included the Posterior Circulation Alberta Stroke Program Early CT Score (PC-ASPECTS), calculated from CT scans. All imaging parameters were reviewed prospectively by board-certified neuroradiologists with over 5 years of experience in acute stroke imaging. In cases of uncertainty, consensus was reached through joint reading. Procedural variables included the pre-hospital triage model (mothership vs drip-and-ship), knowledge of symptom onset time, use of IVT, type of anaesthesia (general, local, or conversion from local to general), onset-to-groin puncture time, number of device passes and rate of first-pass success.

### Outcome evaluation

Functional outcome was assessed using the modified Rankin Scale (mRS) score, evaluated by local stroke neurologists. A favourable outcome was defined as an mRS score of 0–3 at 90 days (±14 days). Mortality was evaluated within 90 days post-stroke to capture fatal outcomes in both patient groups. Symptomatic intracerebral haemorrhage (sICH) was defined as any intracranial haemorrhage identified on neuroimaging within the first 24-h post-stroke, accompanied by a ⩾4-point increase in NIHSS score from baseline. Revascularization success was determined using the modified Thrombolysis in Cerebral Infarction (TICI) classification, with successful recanalization defined as grades 2b, 2c, or 3, indicating substantial reperfusion. Haemorrhagic transformation (HT) was evaluated in accordance with the Heidelberg classification, which includes all haemorrhagic subtypes.^20^

### Study outcomes

The two primary effectiveness outcomes were 90-day favourable mRS (0–3) and the mRS shift after 90 days, assessed through ordinal regression analysis, indicating the shift towards higher mRS scores (worse functional outcome). Secondary effectiveness outcome parameters included successful recanalization (defined as a modified Thrombolysis in Cerebral Infarction (mTICI) score of 2b to 3), 90-day functional independence mRS score (0–2), 90-day excellent outcome mRS score (0–1). Safety outcomes comprised 90-day mortality, sICH and any ICH assessed by intracranial imaging at 24 h. Subgroups were defined according to age (< 80 or ⩾ 80 years), sex, NIHSS at admission (⩽10 and > 10 points), use of IVT and previous use of oral anticoagulation.

### Statistical analysis

Three separate inverse probability weighting (IPW) analyses were performed (aspiration vs stent retriever, aspiration vs combined approach, stent retriever vs combined approach) to balance the baseline characteristics, aiming to reduce the confounding factors on study outcomes. A detailed methodological explanation of the IPW estimation process is available in the Supplemental Methods. In brief, weights were obtained by calculating the probability of being in the groups (aspiration vs stent retriever, aspiration vs combined approach, stent retriever vs combined approach) while controlling for a set of relevant variables that could have influenced the outcome. The weights obtained were then used to balance the baseline covariates, therefore creating a pseudo-population independent of the measured confounders (i.e. pseudo-randomization).^21^ Categorical variables are presented as numbers and percentages, continuous variables as mean and standard deviation or median and interquartile range according to normal distribution. For the primary outcomes, we calculated the risk ratio and risk difference with 95% confidence intervals (CIs) for the 90-day occurrence of mRS score 0–3 between the study groups. The 90-day shift of mRS scores was compared using an ordinal generalized linear model (GLM) and results were presented as Odds ratio (OR) with 95% CIs. For the secondary outcomes, risk ratios (RR) and risk differences (RD) with 95% confidence intervals (CIs) between study groups were calculated for the occurrence of post-procedural favourable TICI score, 90-day functional independence mRS score (0–2), 90-day excellent outcome mRS score (0–1). For all the safety outcomes, we also calculated RR and RD with 95% CIs between the study groups. All outcome analyses were performed in the weighted cohorts. Subgroup analysis of the primary outcome in the weighted cohort was performed in five prespecified subgroups (age [< 80 or ⩾ 80 years], sex, NIHSS at admission [⩽10 and > 10 points], use of IVT and previous use of oral anticoagulation. As the outcomes of the present study were exploratory and there were no assumptions regarding the IPW, a sample size was not prespecified for this analysis. All statistical analyses were performed using R software, version 4.2. Statistical significance was set at a *p* value <0.05.

## Results

A total of 517 patients with BAO who underwent EVT were included in the analysis: 200 were treated with aspiration, 260 with stent retriever and 57 with a combined technique (Supplemental Figure 1). Baseline demographic, clinical and treatment characteristics of the three groups are reported in Table 1. Of note, the combined technique achieved the highest rate of first-pass effect (73.7%) compared to stent retriever (55%) and aspiration alone (41.5%). Overall, after IPW, a good balance was obtained for all major baseline variables of interest (Supplemental Figures 2–4 and Table 1).

**Table 1.** Baseline characteristics.

Variables	Stent retriever (*n* = 260)	Aspiration (*n* = 200)	Combined (*n* = 57)	Stent retriever versus aspiration SMD Weighted*	Combined versus Aspiration SMD weighted*	Combined versus stent retriever SMD weighted*
Demographics						
Age, median (IQR), y	67 (56–77)	70 (57–79.5)	67 (58–79)	0.139	0.097	0.132
Sex, Male, No. (%)	131 (50)	108 (54)	28 (49.1)	0.018	0.041	0.005
Risk factors						
Hypertension, No (%)	147 (57)	106 (53)	31 (54.4)	0.009	0.075	0.021
Hypercholesteromia, No (%)	72 (28)	38 (19)	16 (28.1)	0.031	0.038	0.016
Diabetes, No (%)	47 (18.1)	27 (13.5)	16 (28.1)	0.045	0.018	0.004
Coronary artery disease, No (%)	39 (15)	16 (0.8)	12 (21.1)	0.003	0.008	0.018
Stroke or TIA history, No (%)	26 (10)	26 (13)	9 (15.8)	0.007	0.039	0.011
Frequent alcohol use, No (%)	14 (5.4)	6 (3.0)	9 (15.8)	0.017	0.019	0.009
Current/Former smoker, No (%)	68 (26)	29 (15)	12 (21.1)	0.045	0.002	0.001
Known atrial fibrillation, No (%)	56 (22)	40 (22)	22 (38.6)	0.045	0.009	0.030
AFDAS, No (%)	36 (14)	34 (17)	18 (31.6)	0.011	0.007	0.001
Heart failure, No (%)	22 (8.5)	49 (25)	5 (8.8)	0.020	0.049	0.011
Pre-stroke mRS, median (IQR)	0 (1)	1 (1)	0 (0-1)	0.013	0.243	0.072
Therapy on admission						
Antiplatelet treatment, No (%)	30 (12)	26 (13)	9 (15.8)	0.007	0.046	0.018
Oral anticoagulation, No (%)	30 (12)	9 (4.5)	5 (8.8)	0.003	0.026	0.008
Acute ischaemic stroke characteristics						
Known onset, No (%)	193 (74)	99 (50)	37 (64.9)	0.041	0.044	0.002
NIHSS on admission, median (IQR)	17 (8-23)	16.5 (10-22)	16 (9-25)	0.033	0.137	0.058
PC-ASPECTS, median (IQR)	9 (9-10)	9 (8-10)	9 (9-10)	0.028	0.030	0.052
Procedural features						
Pre-hospital model of care, No (%)				0.039	0.009	0.041
Mothership	136 (52)	63 (32)	32 (56.1)			
Drip-and-ship	124 (48)	137 (68)	25 (43.9)			
Intravenous thrombolysis, No (%)	126 (48)	103 (52)	22 (38.6)	0.054	0.047	0.043
Onset-to-groin time (min), median (IQR)	300 (280–320)	260 (192.5–335)	300 (280–352)	0.041	0.158	0.096
Type of anaesthesia, No (%)				0.069	0.150	0.139
General	165 (63)	27 (14)	28 (49.1)			
Local	26 (10)	31 (16)	16 (28.1)			
Conversion from Local to General	69 (27)	142 (14)	13 (22.8)			
N of passes, median (IQR)	1 (1)	2 (1)	1 (1)	0.055	0.224	0.065
First-pass successful, No (%)	143 (55)	83 (41.5)	42 (73.7)	0.014	0.059	0.040

ASPECTS, Alberta Stroke Program Early CT Score; CA, contact aspiration; GA, general anaesthesia; MT, mechanical thrombectomy; NIHSS, National Institutes of Health Stroke Scale; OAC, oral anticoagulation; SMD, standardized mean difference; SR, stent retriever.

### Stent retriever versus aspiration

**Figure 1.**
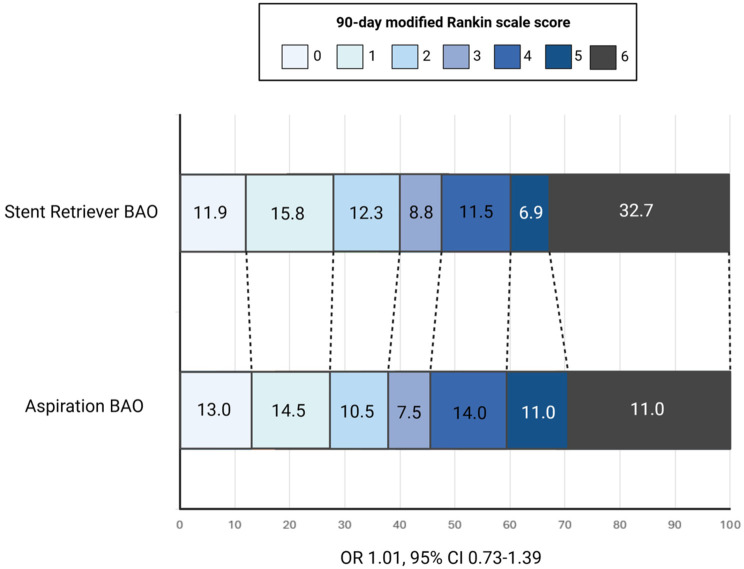
Ninety-day modified Rankin Scale (mRS) shift analysis: Stent retriever versus aspiration as first-line thrombectomy technique. Stacked bar chart displaying the distribution of 90-day mRS scores in patients with basilar artery occlusion (BAO) treated with stent retriever versus aspiration. BAO, basilar artery occlusion; CI, confidence interval; mRS, modified Rankin Scale; OR, odds ratio.

At 90 days, after IPW, favourable mRS was observed in 48.9% of BAO patients treated with stent retriever versus 45.5% in the aspiration group (RR, 1.14; 95% CI, 0.79–1.66; *p* = 0.476), with no significant difference (Table 2). Moreover, at 90 days, there was no difference in the shift of the 90-day mRS scores distribution after IPW (OR 1.01; 95% CI 0.73–1.39; *p* = 0.964) (Figure 1). The rate of successful recanalization was achieved more frequently with stent retriever (RR, 1.86; 95% CI, 1.53–2.26; *p* < 0.001) rather than with aspiration. There were no significant differences regarding the rate of 90-day functional independence and 90-day excellent outcome between the two groups. Finally, no significant differences were observed in rates of 90-day mortality, post-procedural HT and sICH between the two groups after IPW.

**Table 2.** Comparison of study outcomes for Stent retriever versus Aspiration in patients with BAO treated with mechanical thrombectomy.

Study outcomes	Stent retriever (*n* = 260)	Aspiration (*n* = 200)	Statistical metric	Treatment difference [95% CI]	*p* Value
Primary effectiveness outcomes					
90-Day favourable mRS score (0–3), *n* (%)	127 (48.9)	91 (45.5)	Risk ratio	1.14 [0.79 to 1.66]	0.476
			Risk difference (%)	3.35 [−5.85 to12.54]	0.510
90-Day mRS score distribution					
No symptoms (score of 0), *n* (%)	31 (11.9)	26 (13)	Odds ratio	1.01 [0.73 to 1.39]	0.964
Symptoms without any disability (score of 1), *n* (%)	41 (15.8)	29 (14.5)			
Symptoms with mild disability (score of 2), *n* (%)	32 (12.3)	21 (10.5)			
Symptoms with mild-to-moderate disability (score of 3), *n* (%)	23 (8.8)	15 (7.5)			
Symptoms with moderate-to-severe disability (score of 4), *n* (%)	30 (11.5)	28 (14)			
Symptoms with severe disability (score of 5), *n* (%)	18 (6.9)	22 (11)			
Death (score of 6), *n* (%)	85 (32.7)	59 (29.5)			
Secondary outcomes					
Post-procedural favourable TICI score, *n* (%)	181 (69.6)	75 (37.5)	Risk ratio	1.86 [1.53 to 2.26]	**<0.001**
			Risk difference (%)	32.12 [23.38 to 40.85]	**<0.001**
90-Day functional independence mRS score (0–2), *n* (%)	104 (40)	76 (38)	Risk ratio	1.05 [0.84 to 1.33]	0.663
			Risk difference (%)	2 [−6.98 to 10.98]	0.700
90-day excellent outcome mRS score (0–1), *n* (%)	72 (27.7)	55 (27.5)	Risk ratio	1.01 [0.75 to 1.36]	0.964
			Risk difference (%)	0.19 [−8.05 to 8.43]	1.000
Safety outcomes					
90-day death, *n* (%)	85 (32.7)	59 (29.5)	Risk ratio	1.11 [0.84 to 1.46]	0.464
			Risk difference (%)	3.19 [−5.32 to 11.70]	0.479
Post-procedural HT, *n* (%)	64 (24.6)	54 (27)	Risk ratio	0.91 [0.67 to 1.25]	0.562
			Risk difference (%)	−2.38 [−10.46 to 5.69]	0.591
Symptomatic ICH, *n* (%)	30 (11.5)	16 (8)	Risk ratio	1.44 [0.81 to 2.57]	0.210
			Risk difference (%)	3.54 [−1.87 to 8.94]	0.272

CA, contact aspiration; CI, confidence interval; HT, haemorrhagic transformation; mRS, modified Rankin Scale; RD, risk difference; RR, risk ratio; sICH, symptomatic intracranial haemorrhage; SR, stent retriever; TICI, Thrombolysis in Cerebral Infarction.

### Subgroup analysis for the primary outcome in the weighted cohort

Results of subgroup analysis for the primary outcome in the weighted cohort are presented in Supplemental Table 1. Among patients aged < 80 years, the proportion achieving a favourable outcome was comparable between the stent retriever and aspiration groups (51.2% vs 54.7%; RD –3.5%, 95% CI –13.9 to 7; *p* = 0.516). However, in patients aged ⩾80 years, the stent-retriever group demonstrated a significant advantage, with 39.2% achieving mRS 0–3 compared to 18% in the aspiration group (RD 21.2%, 95% CI 4.1 to 38.3; *p* = 0.021). No significant subgroup interactions were observed regarding sex, NIHSS severity (<10 vs ⩾ 10), use of IVT, previous use of oral anticoagulation and presence of AF.

### Combined technique versus aspiration

**Figure 2.**
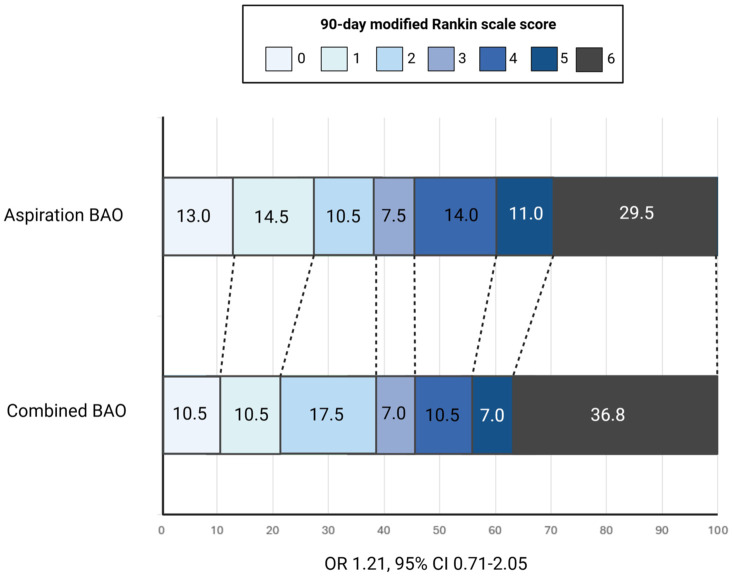
Ninety-day mRS shift analysis: Combined technique versus aspiration. Stacked distribution plot of 90-day mRS outcomes in BAO patients treated with combined stent retriever plus aspiration technique versus aspiration alone. BAO, basilar artery occlusion; CI, confidence interval; mRS, modified Rankin Scale; OR, odds ratio.

At 90 days, after IPW adjustment, a favourable functional outcome (mRS 0–3) was observed in 45.6% of BAO patients treated with the combined technique and in 45.5% of those treated with aspiration alone (RR, 1.00; 95% CI, 0.73–1.38; *p* = 0.988), with no significant difference (Table 3). Similarly, there was no difference in the 90-day mRS distribution shift between groups (OR, 1.21; 95% CI, 0.71–2.05; *p* = 0.479) (Figure 2). The rates of functional independence (mRS 0–2) and excellent outcome (mRS 0–1) did not differ significantly between the combined and aspiration groups. No significant differences were found in the rate of successful recanalization, 90-day mortality or sICH. Notably, the combined technique was associated with a significantly lower rate of post-procedural haemorrhagic transformation (RR, 0.39; 95% CI, 0.20–0.76; *p* = 0.008).

**Table 3.** Comparison of study outcomes for combined versus aspiration in patients with BAO treated with mechanical thrombectomy.

Study outcomes	Combined (*n* = 57)	Aspiration (*n* = 200)	Statistical metric	Treatment difference [95% CI]	*p* Value
Primary effectiveness outcomes					
90-day favourable mRS score (0–3), *n* (%)	26 (45.6)	91 (45.5)	Risk ratio	1.00 [0.73 to 1.38]	0.988
			Risk difference (%)	0.11 [−14.54 to 14.77]	1.000
90-day mRS score distribution					
No symptoms (score of 0), *n* (%)	6 (10.5)	26 (13)	Odds ratio	1.21 [0.71 to 2.05]	0.479
Symptoms without any disability (score of 1), *n* (%)	6 (10.5)	29 (14.5)			
Symptoms with mild disability (score of 2), *n* (%)	10 (17.5)	21 (10.5)			
Symptoms with mild-to-moderate disability (score of 3), *n* (%)	4 (7)	15 (7.5)			
Symptoms with moderate-to-severe disability (score of 4), *n* (%)	6 (10.5)	28 (14)			
Symptoms with severe disability (score of 5), *n* (%)	4 (7)	22 (11)			
Death (score of 6), *n* (%)	21 (36.8)	59 (29.5)			
Secondary outcomes					
Post-procedural favourable TICI score, *n* (%)	24 (42.1)	75 (37.5)	Risk ratio	1.12 [0.79 to 1.60]	0.529
			Risk difference (%)	4.61 [−9.86 to 19.07]	0.540
90-day functional independence mRS score (0-2), *n* (%)	22 (38.6)	76 (38)	Risk ratio	1.02 [0.70 to 1.47]	0.935
			Risk difference (%)	0.60 [−13.72 to 14.91]	1.000
90-day excellent outcome mRS score (0–1), *n* (%)	12 (21.1)	55 (27.5)	Risk ratio	0.77 [0.44 to 1.33]	0.328
			Risk difference (%)	−6.45 [−18.71 to 5.81]	0.394
Safety outcomes					
90-day death, *n* (%)	21 (36.8)	59 (29.5)	Risk ratio	1.25 [0.84 to 1.87]	0.291
			Risk difference (%)	7.34 [−6.69 to 21.37]	0.331
Post-procedural HT, *n* (%)	6 (10.5)	54 (27)	Risk ratio	0.39 [0.18 to 0.86]	0.009
			Risk difference (%)	−16.47 [−26.54 to −6.41]	0.008
Symptomatic ICH, *n* (%)	4 (7)	16 (8)	Risk ratio	0.88 [0.31 to 2.52]	1.000
			Risk difference (%)	−0.98 [−8.61 to 6.64]	1.000

CA, contact aspiration; CI, confidence interval; HT, haemorrhagic transformation; mRS, modified Rankin Scale; RD, risk difference; RR, risk ratio; sICH, symptomatic intracranial haemorrhage; SR, stent retriever; TICI, Thrombolysis in Cerebral Infarction.

### Subgroup analysis for the primary outcome in the weighted cohort

Results of subgroup analysis for the primary outcome in the weighted cohort comparing the combined technique versus aspiration are presented in Supplemental Table 2. No significant subgroup interactions were observed regarding age (<80 vs ⩾ 80 years), sex, NIHSS severity (<10 vs ⩾ 10), use of IVT, previous use of oral anticoagulation and presence of AF.

### Combined technique versus stent retriever

**Figure 3.**
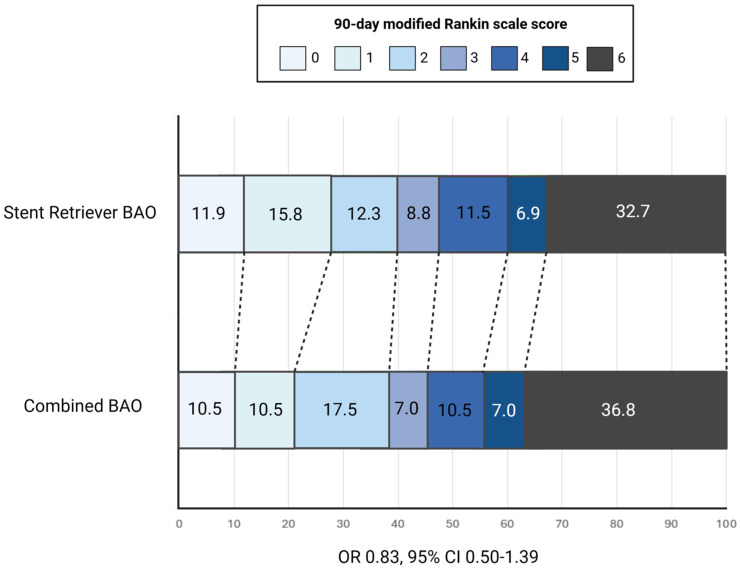
Ninety-day mRS shift analysis: Combined technique versus stent retriever. Ordinal distribution of 90-day mRS scores comparing patients treated with combined thrombectomy versus stent-retriever alone. BAO, basilar artery occlusion; CI, confidence interval; mRS, modified Rankin Scale; OR, odds ratio.

At 90 days, after IPW, a favourable functional outcome (mRS 0–3) was achieved in 45.6% patients treated with combined technique compared to 48.9% of patients treated with aspiration (RR 0.93; 95% CI 0.69–1.27; *p* = 0.6580), indicating no significant difference between the two groups (Table 4). Similarly, no significant difference was observed in the shift analysis of mRS scores (OR 0.83; 95% CI 0.50–1.39; *p* = 0.495) (Figure 3). However, we found a lower rate of successful recanalization (RR, 0.60; 95% CI, 0.44–0.83; *p* < 0.001) and post-procedural HT (RR, 0.43; 95% CI, 0.19–0.94; *p* = 0.020) with the combined approach compared to the stent-retriever. Finally, there were no significant differences in the rates of 90-day functional independence, excellent outcome, 90-day mortality and sICH between the two treatment strategies.

**Table 4.** Comparison of study outcomes for combined versus stent retriever in patients with BAO treated with mechanical thrombectomy.

Study outcomes	Combined (*n* = 57)	Stent retriever (*n* = 260)	Statistical metric	Treatment difference [95% CI]	*p* Value
Primary effectiveness outcomes					
90-day favourable mRS score (0–3), *n* (%)	26 (45.6)	127 (48.9)	Risk ratio	0.93 [0.69 to 1.27]	0.658
			Risk difference (%)	−3.23 [−17.52 to 11.05]	0.664
90-day mRS score distribution					
No symptoms (score of 0), *n* (%)	6 (10.5)	31 (11.9)	Odds ratio	0.83 [0.50 to 1.39]	0.495
Symptoms without any disability (score of 1), *n* (%)	6 (10.5)	41 (15.8)			
Symptoms with mild disability (score of 2), *n* (%)	10 (17.5)	32 (12.3)			
Symptoms with mild-to-moderate disability (score of 3), *n* (%)	4 (7)	23 (8.8)			
Symptoms with moderate-to-severe disability (score of 4), *n* (%)	6 (10.5)	30 (11.5)			
Symptoms with severe disability (score of 5), *n* (%)	4 (7)	18 (6.9)			
Death (score of 6), *n* (%)	21 (36.8)	85 (32.7)			
Secondary outcomes					
Post-procedural favourable TICI score, *n* (%)	24 (42.1)	181 (69.6)	Risk ratio	0.60 [0.44 to 0.83]	<0.001
			Risk difference (%)	−27.51 [−41.49 to −13.53]	<0.001
90-day functional indepedence mRS score (0–2), *n* (%)	22 (38.6)	104 (40)	Risk ratio	0.96 [0.67 to 1.38]	0.845
			Risk difference (%)	−1.40 [−15.37 to 12.57]	0.882
90-day excellent outcome mRS score (0–1), *n* (%)	12 (21.1)	72 (27.7)	Risk ratio	0.77 [0.44 to 1.30]	0.304
			Risk difference (%)	−6.64 [−18.54 to 5.26]	0.326
Safety outcomes					
90-day death, *n* (%)	21 (36.8)	85 (32.7)	Risk ratio	1.13 [0.77 to 1.65]	0.548
			Risk difference (%)	4.15 [−9.61 to 17.91]	0.540
Post-procedural HT, *n* (%)	6 (10.5)	64 (24.6)	Risk ratio	0.43 [0.19 to 0.94]	0.020
			Risk difference (%)	−14.09 [−23.62 to −4.56]	0.021
Symptomatic ICH, *n* (%)	4 (7)	30 (11.5)	Risk ratio	0.61 [0.22 to 1.66]	0.318
			Risk difference (%)	−4.52 [−12.21 to 3.16]	0.477

CA, contact aspiration; CI, confidence interval; HT, haemorrhagic transformation; mRS, modified Rankin Scale; RD, risk difference; RR, risk ratio; sICH, symptomatic intracranial haemorrhage; SR, stent retriever; TICI, Thrombolysis in Cerebral Infarction.

### Subgroup analysis for the primary outcome in the weighted cohort

Results of subgroup analysis for the primary outcome in the weighted cohort comparing the combined technique versus aspiration are presented in Supplemental Table 3. No significant subgroup interactions were observed regarding age (<80 vs ⩾ 80 years), sex, NIHSS severity (<10 vs ⩾ 10), use of IVT, previous use of oral anticoagulation and presence of AF.

### Explorative analysis in BAO patients with NIHSS ⩾ 10

Among patients with BAO and NIHSS ⩾10, baseline characteristics were generally comparable across the stent retriever, aspiration and combined groups, with small weighted standardized mean differences (Supplemental Table 4). Overall, after IPW, a good balance was obtained for all major baseline variables of interest.

Supplemental Table 5 compares study outcomes between stent retriever and aspiration strategies in patients with BAO and NIHSS ⩾10. The proportion of patients achieving favourable functional outcome at 90 days (mRS 0–3) was similar between stent retriever (39.4%) and aspiration (42.9%) groups (RR 1.09, 95% CI 0.84–1.41, *p* = 0.592), with no significant differences in functional independence (mRS 0–2) or excellent outcome (mRS 0–1). The ordinal distribution of 90-day mRS scores also showed no significant shift between approaches (OR 1.08, 95% CI 0.81–1.45, *p* = 0.987). In contrast, aspiration was associated with a substantially higher rate of successful post-procedural reperfusion (TICI ⩾ 2b) compared to stent retriever (67.6% vs 38.8%; RR 1.74, 95% CI 1.36–2.23, *p* < 0.001). Safety outcomes, including 90-day mortality, haemorrhagic transformation and symptomatic intracranial haemorrhage, were comparable across groups, with no statistically significant differences.

Supplemental Table 6 compares outcomes between the stent-retriever strategy and the combined technique. The proportion of patients achieving favourable clinical outcome at 90 days (mRS 0–3) was similar between groups (39.4% vs 40.5%; RR 1.03, 95% CI 0.67–1.59, *p* = 0.909), with similarly comparable rates of functional independence (mRS 0–2) and excellent outcome (mRS 0–1). The distribution of disability levels on the mRS scale showed no significant ordinal shift (OR 1.00, 95% CI 0.69–1.46, *p* = 0.789). Reperfusion success (TICI ⩾2b) was also similar (38.8% vs 32.4%; RR 0.84, 95% CI 0.51–1.39), and no meaningful differences were observed in procedural or safety outcomes, including 90-day mortality and haemorrhagic transformation. However, symptomatic intracranial haemorrhage occurred more frequently in the combined group (18.9% vs 8.1%) (RR 2.33, 95% CI 1.00–5.43, *p* = 0.050).

Finally, Supplemental Table 7 compares outcomes between aspiration and the combined technique. The proportion of patients achieving a favourable 90-day functional outcome (mRS 0–3) was similar between groups (42.9% vs 40.5%; RR 0.94, 95% CI 0.59–1.50), with no meaningful differences in rates of functional independence (mRS 0–2) or excellent outcome (mRS 0–1). The ordinal distribution of mRS scores likewise showed no significant shift across disability levels. In contrast, aspiration achieved markedly higher rates of successful reperfusion (TICI ⩾2b) compared with the combined approach (67.6% vs 32.4%; RR 0.48, 95% CI 0.30–0.76; RD –35.2%, *p* = 0.001). Safety outcomes – including 90-day mortality, haemorrhagic transformation and symptomatic intracranial haemorrhage – did not differ significantly, although the combined group showed numerically higher rates of symptomatic ICH, with wide CIs reflecting its smaller sample size.

## Discussion

In this multicentre cohort of patients with BAO treated with MT, we systematically compared first-line aspiration, stent retriever and combined thrombectomy techniques after rigorous IPW. While no technique demonstrated clear superiority regarding the primary effectiveness outcome, notable differences emerged across secondary endpoints and predefined subgroups. Our analysis showed that the rate of successful recanalization was achieved more frequently with the stent-retriever technique compared to either aspiration or the combined approach. However, the combined technique was associated with the lowest rate of post-procedural haemorrhagic transformation when compared to either the aspiration or stent-retriever technique. Moreover, our subgroup analysis demonstrated that in patients aged ⩾80 years with BAO, the stent retriever had a significant advantage in terms of primary effectiveness when compared to the aspiration technique. Moreover, our exploratory analysis of patients with BAO and severe neurological impairment (NIHSS ⩾10) showed that aspiration consistently achieved higher rates of successful reperfusion compared with both stent-retriever alone and the combined strategy, without translating into superior clinical outcomes. Overall, the study findings suggest that each approach may offer distinct procedural advantages and limitations, which should be considered in treatment decision-making.

Our results are in line with previous studies that did not document a clear impact of the different thrombectomy approaches on 90-day favourable outcome in BAO patients.^14^ Indeed, a post hoc analysis of the BASICS trial compared clinical, technical and safety outcomes of aspiration and stent-retriever thrombectomy as first-line treatment for 158 BAO patients.^22^ This study showed no difference in favourable functional outcome between the two techniques, although the aspiration group was associated with a lower mortality rate at 90 days. Recently, Wischmann et al. analysed data from the German Stroke Registry-Endovascular Treatment (GSR-ET) that included 387 patients with isolated BAO treated with aspiration or stent retriever.^13^ In this study, both aspiration and stent-retriever techniques showed equal efficacy in terms of functional outcome in patients with BAO, although aspiration demonstrated fewer procedure-related complications. However, both studies lacked a three-way comparison including the combined approach, which is increasingly used in clinical practice and may offer distinct advantages that remain to be formally evaluated. Notably, a more comprehensive three-way comparison including the combined approach has been conducted by Abdelrady et al. in the Revascularization via Aspiration or Mechanical thrombectomy in Basilar Occlusion (RAMBO) study.^23^ This study included 128 patients with BAO and did not report significant differences in terms of 90-day functional and safety outcomes among different techniques. However, this study was limited by the potential confounding due to non-random allocation of thrombectomy techniques. In contrast, our analysis adjusted for baseline imbalances using IPW, demonstrated that the combined technique was associated with lower risk of post-procedural HT compared to aspiration and stent retriever. Although sICH rates were low and comparable across the three groups, the presence of haemorrhagic transformation in posterior circulation strokes remains clinically relevant.^24–27^ In this setting, even small volumes of bleeding can exert mass effect within the tightly confined brainstem space, potentially leading to abrupt and catastrophic neurological decline. This risk must be carefully considered when making therapeutic decisions, such as the timing of anticoagulation resumption or the management of other secondary prevention strategies, where the balance between ischaemic recurrence and haemorrhagic complications is particularly delicate.

The observation that 90-day functional outcomes were comparable across the three techniques after adjustment suggests that no single approach is universally superior for BAO and that patient and clot characteristics likely influence device performance. However, the higher RR obtained with stent retrievers may reflect their ability to mechanically engage and extract more organized, fibrin-rich clots.^28^ Conversely, the lower rate of haemorrhagic transformation seen with the combined approach may be related to reduced traction and distal embolization, as aspiration during retrieval can stabilize the clot–device interface and limit endothelial injury.^29,30^ Taken together, these findings support a mechanism-based rather than a “one-technique-fits-all” approach, where clot composition, vessel anatomy and patient profile should inform first-line device selection.

A notable observation in our study is that higher rates of angiographic reperfusion with stent-retriever thrombectomy did not correspond to superior clinical outcomes at 90 days. This apparent dissociation between recanalization and functional recovery has been previously described in patients with BAO undergoing EVT.^31,32^ In BAO, the duration of ischaemia, limited collateral reserve and rapid progression to irreversible tissue injury mean that even successful macrovascular recanalization may occur too late to modify outcome.^31,33^ Furthermore, microvascular no-reflow, distal embolization and reperfusion injury may also blunt the clinical benefit of technically successful thrombectomy.^34^ Thus, recanalization remains a necessary but not sufficient condition for neurological recovery in BAO, emphasizing the importance of minimizing treatment delay and improving strategies to preserve penumbral tissue while revascularization is being pursued.

Recent systematic reviews and meta-analyses comparing aspiration and stent-retriever thrombectomy in BAO have shown nuanced and technique-dependent differences. A large pooled analysis of observational cohorts reported that direct aspiration was associated with higher successful and complete RR, reduced procedure times and lower rates of symptomatic intracranial haemorrhage, whereas functional outcomes and mortality did not differ significantly from stent-retriever approaches.^35^ Similarly, an updated meta-analysis including data up to late 2024 found comparable favourable clinical outcomes and mortality between aspiration and stent retriever, while again noting a lower risk of haemorrhagic complications with aspiration in sensitivity analyses.^36^ These findings highlight that although procedural performance characteristics may differ between techniques, the impact on longer-term outcomes remains uncertain. Importantly, several randomized clinical trials are ongoing, including ANGEL-COAST^37^ and pc-ASTER, which are expected to provide higher-level evidence regarding the comparative benefits of aspiration-first versus stent retriever-first strategies in posterior circulation stroke. Their results will be crucial to define optimal thrombectomy strategy selection in BAO.

The optimal first-line thrombectomy technique in BAO patients remains a matter of debate, and no clear consensus currently exists among neuro-interventionalists. As shown in the international survey by Klein et al., there is substantial heterogeneity in clinical practice, with centres variably adopting stent retriever, contact aspiration, or a combined approach as their initial strategy.^38^ Several factors contribute to this lack of uniformity. First, there is a relative paucity of high-quality, prospective, comparative data specific to BAO, a condition that differs significantly from anterior circulation stroke in terms of anatomy, thrombus characteristics, stroke aetiology and procedural challenges. Second, operator experience and device availability often influence technique selection more than standardized protocols. Third, while the combined approach is increasingly favoured for its potential to maximize first-pass effect and revascularization success, definitive evidence supporting its superiority over single-technique strategies remains limited.^39^ Klein et al. also highlighted that institutional preferences, anecdotal experience and evolving device technology play significant roles in shaping practice patterns. In this context of clinical uncertainty, our study provides timely and relevant data by comparing the three approaches and, importantly, adjusting for confounders through IPW. This methodological rigour allows for a more balanced evaluation of outcomes and offers valuable insights to inform future practice and guideline development.

One of the novel aspects of our study is the inclusion of a predefined subgroup analysis directly comparing the three thrombectomy strategies – aspiration, stent retriever and combined approach – across clinically relevant strata. Such subgroup comparisons are scarce in the existing BAO literature, where most studies report only a two-way comparison between techniques.^40^ Our analysis explored the consistency of treatment effects across age, sex, stroke severity (NIHSS), use of IVT and prior anticoagulation. Interestingly, while most subgroups did not show statistically significant differences in 90-day functional outcomes between techniques, we observed a potentially significant impact in elderly patients (⩾80 years). In this subgroup, patients treated with a stent retriever had a significantly higher rate of favourable outcome compared to those treated with aspiration (risk difference 21.2%, 95% CI 4.1–38.3; *p* = 0.021). No such advantage was seen for the combined technique, which may reflect smaller sample sizes and limited power rather than a true lack of effect. In other subgroups, such as female patients and those with lower NIHSS scores, the combined approach showed numerically better outcomes compared to aspiration or stent-retriever alone, though these did not reach statistical significance. These findings suggest that patient-level characteristics might influence the relative efficacy of different thrombectomy strategies in BAO, highlighting the importance of tailored approaches. However, given the exploratory nature and modest sample sizes, these results should be interpreted with caution and serve as a basis for hypothesis generation in future prospective trials.

This study has several limitations and strengths. Strengths include the multicentre design of the study, large cohort sample and comprehensive three-arm analysis with IPW to balance for confounders. However, our study has several limitations. Although IPW balanced measured confounders, unmeasured factors – such as clot composition, vascular tortuosity, collateral status, stroke aetiology or device generations – may have influenced both technique selection and outcomes. Recanalization quality was assessed by site investigators rather than a central core lab, potentially introducing observer bias. Another limitation is the relatively small sample size of the combined technique group, which may have limited the statistical power to detect significant differences in clinical outcomes or subgroup effects. As a result, findings related to this group – particularly those suggesting a higher first-pass effect or lower haemorrhagic transformation – should be interpreted with caution and warrant confirmation in larger, prospective cohorts. Detailed information on the anatomical location of the basilar artery occlusion (proximal vs distal) was not consistently available across centres and therefore could not be included in the analysis. This may have limited our ability to explore potential effect modification by underlying disease mechanisms. The full TOAST etiological work-up was not consistently captured because patients often returned to their referring centres soon after EVT, and therefore, some uncertainty in stroke mechanism classification may persist. Finally, procedural nuances (e.g., balloon-guide usage, intermediate catheter brands) were not captured, precluding granular technical analysis.

## Conclusion

Our three-way comparison indicates that aspiration, stent retriever and combined techniques yield broadly similar functional outcomes after thrombectomy for BAO, but nuanced differences emerge in elderly outcomes and haemorrhagic safety. These data support continued operator discretion while highlighting subgroups in which specific strategies may confer incremental benefit, thereby informing both current practice and the design of forthcoming randomized trials.

## Supplemental Material

sj-docx-4-tan-10.1177_17562864251410787 – Supplemental material for Aspiration, stent retriever, or combined approach for basilar artery occlusion: a three-way comparative analysis Supplemental material, sj-docx-4-tan-10.1177_17562864251410787 for Aspiration, stent retriever, or combined approach for basilar artery occlusion: a three-way comparative analysis by Muhammad Jaffar, Kazi Ahmed, Samir Abu-Rumeileh, Markus Otto, Lorenzo Barba, Thanh N. Nguyen, Mohamad Abdalkader, Piers Klein, Kyriakos Lobotesis, Mariarosaria Valente, Gian Luigi Gigli, Liqun Zhang, Matteo Foschi, Soma Banerjee, Giovanni Merlino, Robert Simister and Lucio D’Anna in Therapeutic Advances in Neurological Disorders

sj-docx-5-tan-10.1177_17562864251410787 – Supplemental material for Aspiration, stent retriever, or combined approach for basilar artery occlusion: a three-way comparative analysis Supplemental material, sj-docx-5-tan-10.1177_17562864251410787 for Aspiration, stent retriever, or combined approach for basilar artery occlusion: a three-way comparative analysis by Muhammad Jaffar, Kazi Ahmed, Samir Abu-Rumeileh, Markus Otto, Lorenzo Barba, Thanh N. Nguyen, Mohamad Abdalkader, Piers Klein, Kyriakos Lobotesis, Mariarosaria Valente, Gian Luigi Gigli, Liqun Zhang, Matteo Foschi, Soma Banerjee, Giovanni Merlino, Robert Simister and Lucio D’Anna in Therapeutic Advances in Neurological Disorders

sj-docx-6-tan-10.1177_17562864251410787 – Supplemental material for Aspiration, stent retriever, or combined approach for basilar artery occlusion: a three-way comparative analysis Supplemental material, sj-docx-6-tan-10.1177_17562864251410787 for Aspiration, stent retriever, or combined approach for basilar artery occlusion: a three-way comparative analysis by Muhammad Jaffar, Kazi Ahmed, Samir Abu-Rumeileh, Markus Otto, Lorenzo Barba, Thanh N. Nguyen, Mohamad Abdalkader, Piers Klein, Kyriakos Lobotesis, Mariarosaria Valente, Gian Luigi Gigli, Liqun Zhang, Matteo Foschi, Soma Banerjee, Giovanni Merlino, Robert Simister and Lucio D’Anna in Therapeutic Advances in Neurological Disorders

sj-docx-7-tan-10.1177_17562864251410787 – Supplemental material for Aspiration, stent retriever, or combined approach for basilar artery occlusion: a three-way comparative analysis Supplemental material, sj-docx-7-tan-10.1177_17562864251410787 for Aspiration, stent retriever, or combined approach for basilar artery occlusion: a three-way comparative analysis by Muhammad Jaffar, Kazi Ahmed, Samir Abu-Rumeileh, Markus Otto, Lorenzo Barba, Thanh N. Nguyen, Mohamad Abdalkader, Piers Klein, Kyriakos Lobotesis, Mariarosaria Valente, Gian Luigi Gigli, Liqun Zhang, Matteo Foschi, Soma Banerjee, Giovanni Merlino, Robert Simister and Lucio D’Anna in Therapeutic Advances in Neurological Disorders

sj-jpeg-1-tan-10.1177_17562864251410787 – Supplemental material for Aspiration, stent retriever, or combined approach for basilar artery occlusion: a three-way comparative analysis Supplemental material, sj-jpeg-1-tan-10.1177_17562864251410787 for Aspiration, stent retriever, or combined approach for basilar artery occlusion: a three-way comparative analysis by Muhammad Jaffar, Kazi Ahmed, Samir Abu-Rumeileh, Markus Otto, Lorenzo Barba, Thanh N. Nguyen, Mohamad Abdalkader, Piers Klein, Kyriakos Lobotesis, Mariarosaria Valente, Gian Luigi Gigli, Liqun Zhang, Matteo Foschi, Soma Banerjee, Giovanni Merlino, Robert Simister and Lucio D’Anna in Therapeutic Advances in Neurological Disorders

sj-jpeg-2-tan-10.1177_17562864251410787 – Supplemental material for Aspiration, stent retriever, or combined approach for basilar artery occlusion: a three-way comparative analysis Supplemental material, sj-jpeg-2-tan-10.1177_17562864251410787 for Aspiration, stent retriever, or combined approach for basilar artery occlusion: a three-way comparative analysis by Muhammad Jaffar, Kazi Ahmed, Samir Abu-Rumeileh, Markus Otto, Lorenzo Barba, Thanh N. Nguyen, Mohamad Abdalkader, Piers Klein, Kyriakos Lobotesis, Mariarosaria Valente, Gian Luigi Gigli, Liqun Zhang, Matteo Foschi, Soma Banerjee, Giovanni Merlino, Robert Simister and Lucio D’Anna in Therapeutic Advances in Neurological Disorders

sj-jpeg-3-tan-10.1177_17562864251410787 – Supplemental material for Aspiration, stent retriever, or combined approach for basilar artery occlusion: a three-way comparative analysis Supplemental material, sj-jpeg-3-tan-10.1177_17562864251410787 for Aspiration, stent retriever, or combined approach for basilar artery occlusion: a three-way comparative analysis by Muhammad Jaffar, Kazi Ahmed, Samir Abu-Rumeileh, Markus Otto, Lorenzo Barba, Thanh N. Nguyen, Mohamad Abdalkader, Piers Klein, Kyriakos Lobotesis, Mariarosaria Valente, Gian Luigi Gigli, Liqun Zhang, Matteo Foschi, Soma Banerjee, Giovanni Merlino, Robert Simister and Lucio D’Anna in Therapeutic Advances in Neurological Disorders

## Declarations

**Ethics approval and consent to participate:** This study has obtained approval from the UK Health Regulator Authority (Health Regulator Authority Reference No.: 275260). The study has also received confirmation of capacity and capability from the Imperial College Healthcare NHS Trust (170843). Informed consent was not a legal requirement, as the research was carried out using data collected as part of routine care and any researchers outside the direct care team only had access to anonymized data. The study was conducted in accordance with the recommendations for physicians involved in research on human subjects adopted by the 18th World Medical Assembly, Helsinki 1964 and later revisions. The corresponding author had full access to all the data in the study and takes responsibility for its integrity and the data analysis.

**Consent for publication:** The requirement for consent for publication was waived by the relevant ethics committee, as the study used anonymised data only.

**Availability of data and materials:** Data available upon reasonable request.
