# Supplementary material for: Aspiration, stent retriever, or combined approach for basilar artery occlusion: a three-way comparative analysis
Source: Ther Adv Neurol Disord. 2026 Jan 29;19:17562864251410787. doi: 10.1177/17562864251410787 (PMC12855751; doi:10.1177/17562864251410787)
Supplement: sj-docx-4-tan-10.1177_17562864251410787 – Supplemental material for Aspiration, stent retriever, or combined approach for basilar artery occlusion: a three-way comparative analysis [file sj-docx-4-tan-10.1177_17562864251410787.docx]

**Supplemental Table 1. Subgroup analysis for 90-day favorable mRS score (0-3) for Stent retriever vs Aspiration in patients with BAO treated with mechanical thrombectomy.**

|  | **No. of patients** | **Stent retriever** | **Aspiration** | **Risk difference* (%) [95% CI]** | **P value** |
| --- | --- | --- | --- | --- | --- |
| **Age, years** |  |  |  |  |  |
| **<80** | 359 | 107/209 (51.2) | 82/150 (54.7) | -3.5 [-13.9 to 7] | 0.516 |
| **≥80** | 101 | 20/51 (39.2) | 9/50 (18) | 21.2 [4.1 to 38.3] | **0.021** |
| **Sex** |  |  |  |  |  |
| **Female** | 221 | 67/129 (51.9) | 39/92 (42.4) | 9.5 [-0.04 to 22.8] | 0.162 |
| **Male** | 239 | 60/131 (45.8) | 52/108 (48.1) | -2.3 [-15.1 to 10.4] | 0.717 |
| **NIHSS** |  |  |  |  |  |
| **<10** | 118 | 49/78 (62.8) | 28/40 (70) | -7.2 [-25 to 10.6] | 0.439 |
| **≥10** | 342 | 78/182 (42.9) | 63/160 (39.4) | 3.5 [-7 to 13.9] | 0.514 |
| **Intravenous thrombolysis** |  |  |  |  |  |
| **No** | 231 | 58/134 (43.3) | 34/97 (35.1) | 8.2 [-4.4 to 20.9] | 0.208 |
| **Yes** | 229 | 69/126 (54.8) | 57/103 (55.3) | -0.6 [-13.5 to 12.4] | 0.930 |
| **Previous use of Oral anticoagulation** |  |  |  |  |  |
| **No** | 421 | 109/230 (47.4) | 85/191 (44.5) | 2.9 [-6.7 to 12.4] | 0.554 |
| **Yes** | 39 | 18/30 (60) | 6/9 (66.7) | -6.7 [-4.2 to 28.8] | 0.719 |
| **Atrial fibrillation** |  |  |  |  |  |
| **No** | 294 | 80/168 (47.6) | 55/126 (43.7) | 3.9 [-7.6 to 15.4] | 0.500 |
| **Yes** | 166 | 30/92 (32.6) | 18/74 (24.3) | 8.3 [-5.4 to 22.0] | 0.243 |

**Supplemental Table 2. Subgroup analysis for 90-day favorable mRS score (0-3) for Combined vs Aspiration in patients with BAO treated with mechanical thrombectomy.**

|  | **No. of patients** | **Combined** | **Aspiration** | **Risk difference* (%) [95% CI]** | **P value** |
| --- | --- | --- | --- | --- | --- |
| **Age, years** |  |  |  |  |  |
| **<80** | 193 | 22/43 (51.2) | 82/150 (54.7) | -3.5 [-20.4 to 13.4] | 0.684 |
| **≥80** | 64 | 4/14 (28.6) | 9/50 (18) | 10.6 [-15.4 to 36.5] | 0.389 |
| **Sex** |  |  |  |  |  |
| **Female** | 121 | 17/29 (58.6) | 39/92 (42.4) | 16.23 [-4.34 to 36.80] | 0.140 |
| **Male** | 136 | 9/28 (32.1) | 52/108 (48.1) | -16.01 [-35.70 to 3.69] | 0.142 |
| **NIHSS** |  |  |  |  |  |
| **<10** | 60 | 11/20 (55) | 28/40 (70) | -15 [-41.02 to 11.02] | 0.268 |
| **≥10** | 197 | 15/37 (40.5) | 63/160 (39.4) | 1.17 [-16.37 to 18.70] | 1.000 |
| **Intravenous thrombolysis** |  |  |  |  |  |
| **No** | 132 | 14/35 (40) | 34/97 (35.1) | 4.95 [-13.86 to 23.75] | 0.683 |
| **Yes** | 125 | 12/22 (54.6) | 57/103 (55.3) | -0.79 [-23.71 to 22.12] | 1.000 |
| **Previous use of Oral anticoagulation** |  |  |  |  |  |
| **No** | 243 | 22/52 (42.3) | 85/191 (44.5) | -2.19 [-17.36 to 12.97] | 0.683 |
| **Yes** | 14 | 4/5 (80) | 6/9 (66.7) | 13.33 [-33.33 to 60] | 1.000 |
| **Atrial fibrillation** |  |  |  |  |  |
| **No** | 143 | 7/17 (41.2) | 55/126 (43.7) | -2.5 [-27.4 to 22.5] | 0.846 |
| **Yes** | 114 | 12/40 (30.0) | 18/74 (24.3) | 5.7 [-11.5 to 22.9] | 0.517 |

**Supplemental Table 3. Subgroup analysis for 90-day favorable mRS score (0-3) for Combined vs Stent retriever in patients with BAO treated with mechanical thrombectomy.**

|  | **No. of patients** | **Combined** | **Stent retriever** | **Risk difference* (%) [95% CI]** | **P value** |
| --- | --- | --- | --- | --- | --- |
| **Age, years** |  |  |  |  |  |
| **<80** | 252 | 22/43 (51.2) | 107/209 (51.2) | 0 [-16.4 to 16.4] | 0.996 |
| **≥80** | 65 | 4/14 (28.6) | 20/51 (39.2) | -10.6 [-37.8 to 16.6] | 0.467 |
| **Sex** |  |  |  |  |  |
| **Female** | 158 | 17/29 (58.6) | 67/129 (51.9) | 6.7 [-13.2 to 26.6] | 0.515 |
| **Male** | 159 | 9/28 (32.1) | 60/131 (45.8) | -13.7 [-32.9 to 5.6] | 0.189 |
| **NIHSS** |  |  |  |  |  |
| **<10** | 98 | 11/20 (55) | 49/78 (62.8) | -7.8 [-32.1 to 16.5] | 0.523 |
| **≥10** | 217 | 15/37 (40.5) | 78/182 (42.9) | -2.3 [-19.7 to 15.1] | 0.795 |
| **Intravenous thrombolysis** |  |  |  |  |  |
| **No** | 169 | 14/35 (40) | 58/134 (43.3) | -3.3 [-21.6 to 15] | 0.727 |
| **Yes** | 148 | 12/22 (54.6) | 69/126 (54.8) | -0.2 [-22.8 to 22.3] | 0.985 |
| **Previous use of Oral anticoagulation** |  |  |  |  |  |
| **No** | 282 | 22/52 (42.3) | 109/230 (47.4) | -5.1 [-20 to 9.8] | 0.507 |
| **Yes** | 35 | 4/5 (80) | 18/30 (60) | 20 [-19.2 to 59.2] | 0.403 |
| **Atrial fibrillation** |  |  |  |  |  |
| **No** | 185 | 7/17 (41.2) | 80/168 (47.6) | -6.4 [-31.0 to 18.1] | 0.800 |
| **Yes** | 132 | 12/40 (30.0) | 30/92 (32.6) | -2.6 [-19.7 to 14.5] | 0.840 |
