# Supplementary material for: Aspiration, stent retriever, or combined approach for basilar artery occlusion: a three-way comparative analysis
Source: Ther Adv Neurol Disord. 2026 Jan 29;19:17562864251410787. doi: 10.1177/17562864251410787 (PMC12855751; doi:10.1177/17562864251410787)
Supplement: sj-docx-5-tan-10.1177_17562864251410787 – Supplemental material for Aspiration, stent retriever, or combined approach for basilar artery occlusion: a three-way comparative analysis [file sj-docx-5-tan-10.1177_17562864251410787.docx]

**SUPPLEMENTARY METHODS**

**Inverse probability weighting calculation**

Firstly, we calculated the probability of assignment to a mechanical thrombectomy technique adjusting for a set of pre-defined covariates (i.e. propensity score), specifically: age, sex, presence of hypertension, hypercholesterolemia, atrial dysfunction, heart failure, coronary artery disease, smoking, diabetes, alcohol use, previous stroke, pre-stroke mRS, antiplatelet agents, anticoagulants, known stroke onset, pre-stroke model of care, use of intravenous thrombolysis, onset to groin time, baseline NIHSS, type of anesthesia, ASPECTS score, first-pass successful, number of passes. Subsequently, stabilized weights were obtained dividing the crude probability of the observed exposure by the propensity scores, and weight balance was assessed with standardized mean differences (SMD), by considering a value <0.2 an acceptable difference and <0.1 a negligible difference.
