# Supplementary material for: Aspiration, stent retriever, or combined approach for basilar artery occlusion: a three-way comparative analysis
Source: Ther Adv Neurol Disord. 2026 Jan 29;19:17562864251410787. doi: 10.1177/17562864251410787 (PMC12855751; doi:10.1177/17562864251410787)
Supplement: sj-docx-6-tan-10.1177_17562864251410787 – Supplemental material for Aspiration, stent retriever, or combined approach for basilar artery occlusion: a three-way comparative analysis [file sj-docx-6-tan-10.1177_17562864251410787.docx]

**Supplemental Table 4. Baseline characteristics for patient with BAO and NIHSS ≥ 10.**

|  | **Stent retriever**  **(n=160)** | **Aspiration**  **(n=182)** | **Combined**  **(n=37)** | **Stent retriever vs Aspiration**  **SMD**  **Weighted*** | **Combined vs Aspiration**  **SMD**  **weighted*** | **Combined vs Stent retriever SMD**  **weighted*** |
| --- | --- | --- | --- | --- | --- | --- |
| **Demographics** |  |  |  |  |  |  |
| Age, median (IQR), y | 73 (60-80) | 68 (57-77) | 68 (60-79) | 0.0699 | 0.0569 | 0.0999 |
| Sex, Male, No. (%) | 89 (56) | 92 (51) | 18 (49) | 0.0027 | 0.0022 | 0.0033 |
| **Risk factors** |  |  |  |  |  |  |
| Hypertension, No (%) | 90 (56) | 100 (55) | 21 (57) | 0.0232 | 0.0105 | 0.0218 |
| Hypercholesteromia, No (%) | 12 (7) | 27 (15) | 10 (27) | 0.0203 | 0.0045 | 0.0210 |
| Diabetes, No (%) | 24 (15) | 32 (18) | 9 (24) | 0.0272 | 0.0551 | 0.0101 |
| Coronary artery disease, No (%) | 12 (8) | 27 (15) | 10 (27) | 0.0395 | 0.0210 | 0.0395 |
| Stroke or TIA history, No (%) | 22 (14) | 13 (7) | 6 (16) | 0.0096 | 0.0128 | 0.0101 |
| Frequent alcohol use, No (%) | 4 (3) | 9 (5) | 5 (14) | 0.0377 | 0.0232 | 0.0045 |
| Current/Former smoker, No (%) | 19 (12) | 51 (28) | 9 (24) | 0.0724 | 0.0203 | 0.0096 |
| Known atrial fibrillation, No (%) | 37 (23) | 30 (17) | 14 (38) | 0.0377 | 0.0395 | 0.0045 |
| AFDAS, No (%) | 31 (19) | 23 (13) | 11 (30) | 0.0724 | 0.0096 | 0.0375 |
| Heart failure, No (%) | 46 (29) | 16 (9) | 4 (11) | 0.0551 | 0.0377 | 0.0232 |
| **Pre-stroke mRS, median (IQR)** | 1 (0-2) | 0 (0-1) | 0 (0-1) | 0.0101 | 0.0395 | 0.0218 |
| **Therapy on admission** |  |  |  |  |  |  |
| Antiplatelet treatment, No (%) | 25 (16) | 17 (9) | 8 (22) | 0.0003 | 0.0551 | 0.0003 |
| Oral anticoagulation, No (%) | 5 (3) | 18 (10) | 3 (8) | 0.0218 | 0.0218 | 0.0218 |
| **Acute ischemic stroke characteristics** |  |  |  |  |  |  |
| Known onset, No (%) | 69 (43) | 136 (75) | 24 (65) | 0.0218 | 0.0218 | 0.0395 |
| NIHSS on admission, median (IQR) | 18 (15-23) | 21 (16-26) | 23 (17-27) | 0.0210 | 0.0377 | 0.0375 |
| PC-ASPECTS, median (IQR) | 10 (9-10) | 9 (8-10) | 9 (9-10) | 0.0218 | 0.0724 | 0.0991 |
| **Procedural features** |  |  |  |  |  |  |
| Pre-hospital model of care, No (%) |  |  |  | 0.0038 | 0.0055 | 0.0230 |
| Mothership | 50 (31) | 92 (51) | 20 (54) |  |  |  |
| Drip-and-ship | 110 (69) | 90 (49) | 17 (46) |  |  |  |
| Intravenous thrombolysis, No (%) | 81 (51) | 92 (51) | 14 (38) | 0.0582 | 0.0045 | 0.0991 |
| Onset to groin time (min), median (IQR) | 252 (190-300) | 300 (300-300) | 300 (300-395) | 0.0375 | 0.0551 | 0.0128 |
| Type of anesthesia, No (%) |  |  |  | 0.0353 | 0.0367 | 0.0325 |
| General | 112 (70) | 55 (30) |  |  |  |  |
| Local | 23 (14) | 109 (60) |  |  |  |  |
| Conversion from Local to General | 25 (16) | 18 (10) |  |  |  |  |
| N of passes, median (IQR) | 2 (1-2) | 1 (1-2) | 1 (1-2) | 0.0991 | 0.0351 | 0.0303 |
| First pass successful, No (%) | 81 (51) | 109 (60) | 26 (70) | 0.0128 | 0.0233 | 0.0222 |

*Abbreviations MT, mechanical thrombectomy; SR, stent retriever; CA, contact aspiration; NIHSS, National Institutes of Health Stroke Scale; ASPECTS, Alberta Stroke Program Early CT Score; GA, general anesthesia; OAC, oral anticoagulation; SMD, standardized mean difference.*

**Supplemental Table 5. Comparison of study outcomes for Stent retriever vs Aspiration in patients with BAO treated with mechanical thrombectomy in patients with NIHSS ≥ 10.**

|  | **Stent retriever**  **(n=160)** | **Aspiration**  **(n=182)** | **Statistical metric** | **Treatment difference [95% CI]** | **P value** |
| --- | --- | --- | --- | --- | --- |
| ***Primary effectiveness outcomes*** |  |  |  |  |  |
| 90-day favorable mRS score (0-3), n (%) | 63 (39.4) | 78 (42.9) | **Risk ratio** | 1.09 [0.84-1.41] | 0.592 |
|  |  |  | **Risk difference (%)** | 3.5 [-7.2-14.3] | 0.595 |
| 90-day mRS score distribution |  |  |  |  |  |
| No symptoms (score of 0), n (%) | 20 (12.5) | 22 (12.1) | **Odds ratio** | 1.08 [0.81-1.45] | 0.987 |
| Symptoms without any disability (score of 1), n (%) | 18 (11.2) | 24 (13.2) |  |  |  |
| Symptoms with mild disability (score of 2), n (%) | 14 (8.8) | 19 (10.4) |  |  |  |
| Symptoms with mild-to-moderate disability (score of 3), n (%) | 11 (6.9) | 13 (7.1) |  |  |  |
| Symptoms with moderate-to-severe disability (score of 4), n (%) | 26 (16.2) | 22 (12.1) |  |  |  |
| Symptoms with severe disability (score of 5), n (%) | 19 (11.9) | 17 (9.3) |  |  |  |
| Death (score of 6), n (%) | 52 (32.5) | 65 (35.7) |  |  |  |
| ***Secondary outcomes*** |  |  |  |  |  |
| Post-procedural favorable TICI score, n (%) | 62 (38.8) | 123 (67.6) | **Risk ratio** | 1.74 [1.36-2.23] | **<0.001** |
|  |  |  | **Risk difference (%)** | 28.8 [17.5-40.1] | **<0.001** |
| 90-day functional indepedence mRS score (0-2), n (%) | 52 (32.5) | 65 (35.7) | **Risk ratio** | 1.10 [0.81-1.51] | 0.334 |
|  |  |  | **Risk difference (%)** | 3.2 [-8.7-15.1] | 0.341 |
| 90-day excellent outcome mRS score (0-1), n (%) | 38 (24.2) | 46 (25.3) | **Risk ratio** | 1.05 [0.74-1.50] | 0.702 |
|  |  |  | **Risk difference (%)** | 1.1 [-8.9-11.1] | 0.703 |
| ***Safety outcomes*** |  |  |  |  |  |
| 90-day death, n (%) | 52 (32.5) | 65 (35.7) | **Risk ratio** | 1.10 [0.82-1.49] | 0.535 |
|  |  |  | **Risk difference (%)** | 3.2 [-9.6-16.0] | 0.531 |
| Post-procedural HT, n (%) | 40 (25) | 47 (25.8) | **Risk ratio** | 1.03 [0.73-1.44] | 0.860 |
|  |  |  | **Risk difference (%)** | 0.8 [-10.3-12] | 0.309 |
| Symptomatic ICH, n (%) | 13 (8.1) | 19 (10.4) | **Risk ratio** | 1.29 [0.66-2.51] | 0.378 |
|  |  |  | **Risk difference (%)** | 2.3 [-4.5-9.0] | 0.361 |

*Abbreviations: SR, stent retriever; CA, contact aspiration; mRS, modified Rankin Scale; TICI, Thrombolysis in Cerebral Infarction; sICH, symptomatic intracranial hemorrhage; HT, hemorrhagic transformation; RR, risk ratio; RD, risk difference; CI, confidence interval.*

**Supplemental Table 6. Comparison of study outcomes for Stent retriever vs Combined in patients with BAO treated with mechanical thrombectomy in patients with NIHSS ≥ 10.**

|  | **Stent retriever**  **(n=160)** | **Combined**  **(n=37)** | **Statistical metric** | **Treatment difference [95% CI]** | **P value** |
| --- | --- | --- | --- | --- | --- |
| ***Primary effectiveness outcomes*** |  |  |  |  |  |
| 90-day favorable mRS score (0-3), n (%) | 63 (39.4) | 15 (40.5) | **Risk ratio** | 1.03 [0.67-1.59] | 0.909 |
|  |  |  | **Risk difference (%)** | 1.2 [-16.4-18.7] | 0.900 |
| 90-day mRS score distribution |  |  |  |  |  |
| No symptoms (score of 0), n (%) | 20 (12.5) | 5 (13.5) | **Odds ratio** | 1.00 [0.69-1.46] | 0.789 |
| Symptoms without any disability (score of 1), n (%) | 18 (11.2) | 2 (5.4) |  |  |  |
| Symptoms with mild disability (score of 2), n (%) | 14 (8.8) | 6 (16.2) |  |  |  |
| Symptoms with mild-to-moderate disability (score of 3), n (%) | 11 (6.9) | 2 (5.4) |  |  |  |
| Symptoms with moderate-to-severe disability (score of 4), n (%) | 26 (16.2) | 6 (16.2) |  |  |  |
| Symptoms with severe disability (score of 5), n (%) | 19 (11.9) | 3 (8.1) |  |  |  |
| Death (score of 6), n (%) | 52 (32.5) | 13 (35.1) |  |  |  |
| ***Secondary outcomes*** |  |  |  |  |  |
| Post-procedural favorable TICI score, n (%) | 62 (38.8) | 12 (32.4) | **Risk ratio** | 0.84 [0.51-1.39] | 0.490 |
|  |  |  | **Risk difference (%)** | -6.4 [-23.6-10.8] | 0.470 |
| 90-day functional indepedence mRS score (0-2), n (%) | 52 (32.5) | 13 (35.1) | **Risk ratio** | 1.08 [0.65-1.80] | 0.760 |
|  |  |  | **Risk difference (%)** | 2.6 [-14.5-19.8] | 0.769 |
| 90-day excellent outcome mRS score (0-1), n (%) | 38 (24.2) | 7 (18.9) | **Risk ratio** | 0.78 [0.35-1.70] | 0.530 |
|  |  |  | **Risk difference (%)** | -5.3 [-18.1-7.5] | 0.410 |
| ***Safety outcomes*** |  |  |  |  |  |
| 90-day death, n (%) | 52 (32.5) | 13 (35.1) | **Risk ratio** | 1.08 [0.65-1.80] | 0.760 |
|  |  |  | **Risk difference (%)** | 2.6 [-20.5-27.7] | 0.770 |
| Post-procedural HT, n (%) | 40 (25) | 11 (29.7) | **Risk ratio** | 1.19 [0.65-2.09] | 0.570 |
|  |  |  | **Risk difference (%)** | 4.7 [-11.5-20.9] | 0.579 |
| Symptomatic ICH, n (%) | 13 (8.1) | 7 (18.9) | **Risk ratio** | 2.33 [1.00-5.43] | 0.050 |
|  |  |  | **Risk difference (%)** | 10.8 [-2.5-24.1] | 0.110 |

*Abbreviations: SR, stent retriever; CA, contact aspiration; mRS, modified Rankin Scale; TICI, Thrombolysis in Cerebral Infarction; sICH, symptomatic intracranial hemorrhage; HT, hemorrhagic transformation; RR, risk ratio; RD, risk difference; CI, confidence interval.*

**Supplemental Table 7. Comparison of study outcomes for Aspiration vs Combined in patients with BAO treated with mechanical thrombectomy in patients with NIHSS ≥ 10.**

|  | **Aspiration**  **(n=182)** | **Combined**  **(n=37)** | **Statistical metric** | **Treatment difference [95% CI]** | **P value** |
| --- | --- | --- | --- | --- | --- |
| ***Primary effectiveness outcomes*** |  |  |  |  |  |
| 90-day favorable mRS score (0-3), n (%) | 78 (42.9) | 15 (40.5) | **Risk ratio** | 0.94 [0.59-1.50] | 0.800 |
|  |  |  | **Risk difference (%)** | -2.4 [-20.2-15.4] | 0.879 |
| 90-day mRS score distribution |  |  |  |  |  |
| No symptoms (score of 0), n (%) | 22 (12.1) | 5 (13.5) | **Odds ratio** | 1.06 [0.72-1.57] | 0.654 |
| Symptoms without any disability (score of 1), n (%) | 24 (13.2) | 2 (5.4) |  |  |  |
| Symptoms with mild disability (score of 2), n (%) | 19 (10.4) | 6 (16.2) |  |  |  |
| Symptoms with mild-to-moderate disability (score of 3), n (%) | 13 (7.1) | 2 (5.4) |  |  |  |
| Symptoms with moderate-to-severe disability (score of 4), n (%) | 22 (12.1) | 6 (16.2) |  |  |  |
| Symptoms with severe disability (score of 5), n (%) | 17 (9.3) | 3 (8.1) |  |  |  |
| Death (score of 6), n (%) | 65 (35.7) | 13 (35.1) |  |  |  |
| ***Secondary outcomes*** |  |  |  |  |  |
| Post-procedural favorable TICI score, n (%) | 123 (67.6) | 12 (32.4) | **Risk ratio** | 0.48 [0.30-0.76] | **0.001** |
|  |  |  | **Risk difference (%)** | -35.2 [-52.3-18.1] | **0.001** |
| 90-day functional indepedence mRS score (0-2), n (%) | 65 (35.7) | 13 (35.1) | **Risk ratio** | 0.98 [0.59-1.65] | 0.950 |
|  |  |  | **Risk difference (%)** | -0.6 [-18.6-17.4] | 0.899 |
| 90-day excellent outcome mRS score (0-1), n (%) | 46 (25.3) | 7 (18.9) | **Risk ratio** | 0.75 [0.37-1.46] | 0.399 |
|  |  |  | **Risk difference (%)** | -6.4 [-19.7-6.8] | 0.703 |
| ***Safety outcomes*** |  |  |  |  |  |
| 90-day death, n (%) | 65 (35.7) | 13 (35.1) | **Risk ratio** | 0.98 [0.62-1.54] | 0.940 |
|  |  |  | **Risk difference (%)** | -0.6 [-20.1-18.9] | 0.566 |
| Post-procedural HT, n (%) | 47 (25.8) | 11 (29.7) | **Risk ratio** | 1.15 [0.68-1.93] | 0.654 |
|  |  |  | **Risk difference (%)** | 3.9 [-11.9-19.7] | 0.564 |
| Symptomatic ICH, n (%) | 19 (10.4) | 7 (18.9) | **Risk ratio** | 1.82 [0.85-3.88] | 0.129 |
|  |  |  | **Risk difference (%)** | 8.5 [-2.4-19.3] | 0.344 |

*Abbreviations: SR, stent retriever; CA, contact aspiration; mRS, modified Rankin Scale; TICI, Thrombolysis in Cerebral Infarction; sICH, symptomatic intracranial hemorrhage; HT, hemorrhagic transformation; RR, risk ratio; RD, risk difference; CI, confidence interval.*

**Supplemental Table 8. Procedural rescue maneuvers and periprocedural medication use.**

|  | **Stent retriever (n=260)** | **Aspiration (n=200)** | **Combined (n=57)** |
| --- | --- | --- | --- |
| **Intracranial angioplasty and/or permanent stent**, n (%) | 21 (8) | 0 (0) | 5 (8.8) |
| **Intra-procedural GP IIb/IIIa inhibitor use**, n (%) | 13 (5) | 6 (3) | 7 (12) |
| **Immediate dual antiplatelet therapy post-procedure**, n (%) | 18 (7) | 0 (0) | 4 (7) |
