# Supplementary figures and images for: Aspiration, stent retriever, or combined approach for basilar artery occlusion: a three-way comparative analysis
Source: Ther Adv Neurol Disord. 2026 Jan 29;19:17562864251410787. doi: 10.1177/17562864251410787 (PMC12855751; doi:10.1177/17562864251410787)

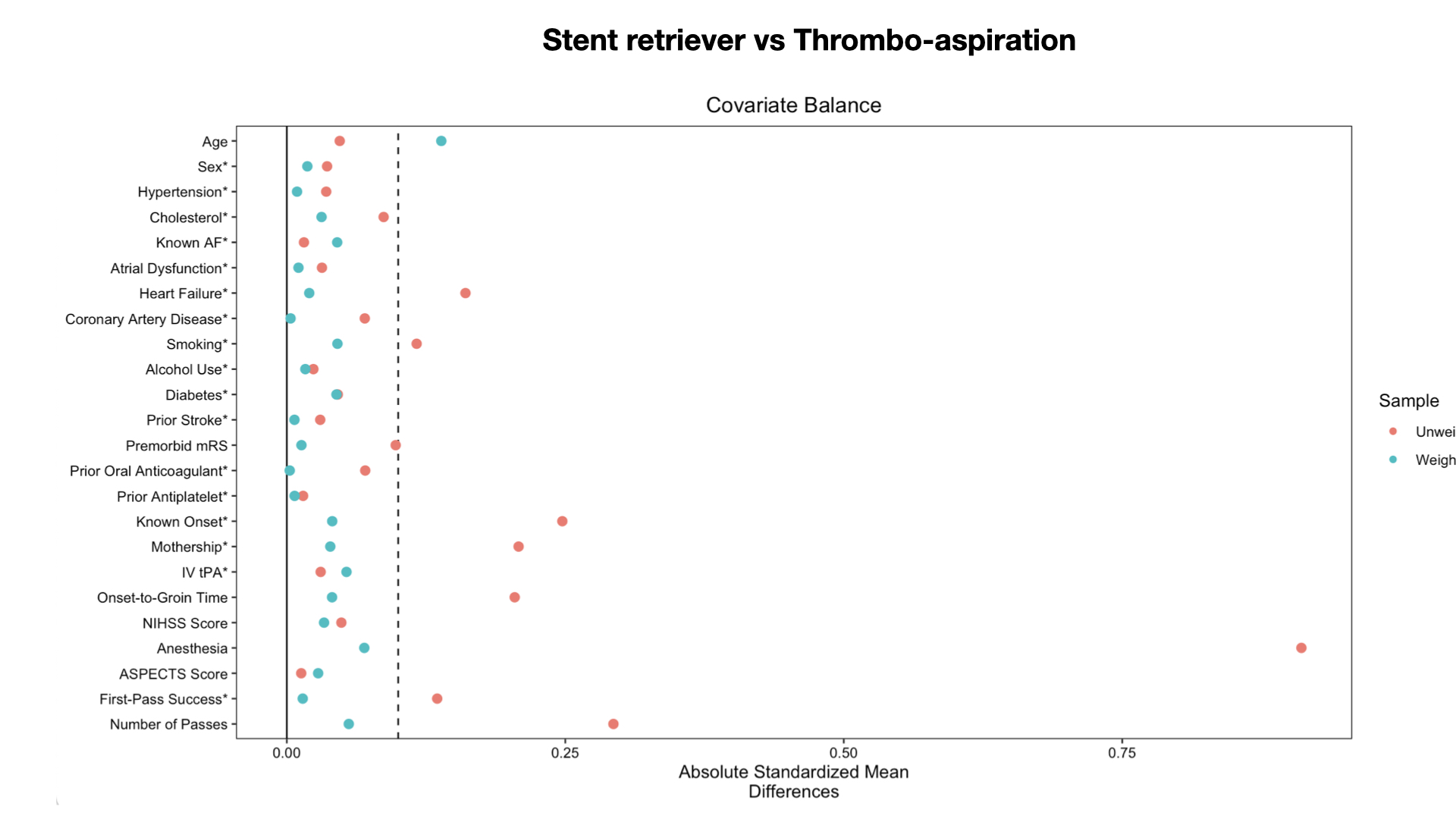

Supplement: sj-jpeg-1-tan-10.1177_17562864251410787 – Supplemental material for Aspiration, stent retriever, or combined approach for basilar artery occlusion: a three-way comparative analysis [file sj-jpeg-1-tan-10.1177_17562864251410787.jpeg]

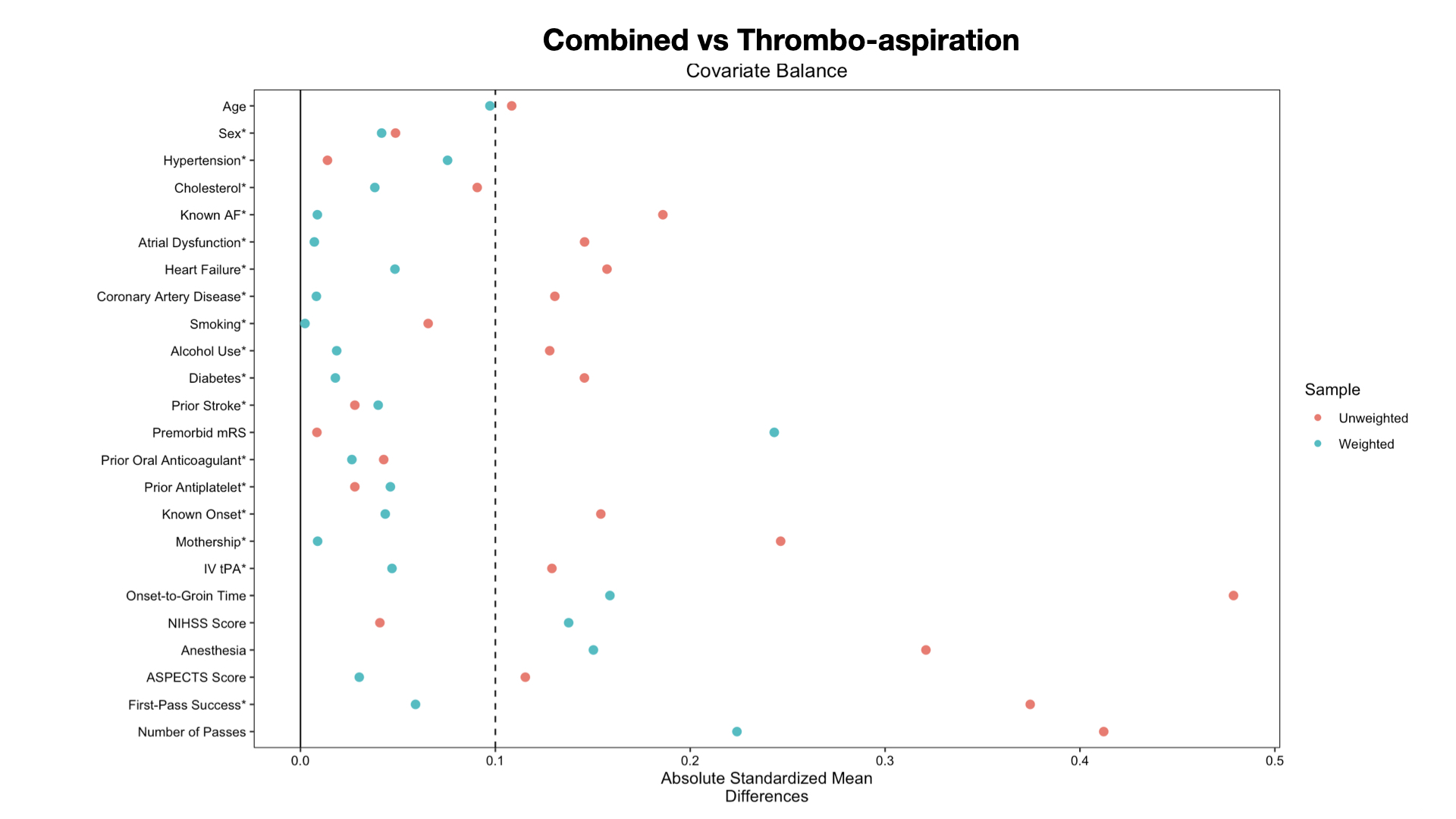

Supplement: sj-jpeg-2-tan-10.1177_17562864251410787 – Supplemental material for Aspiration, stent retriever, or combined approach for basilar artery occlusion: a three-way comparative analysis [file sj-jpeg-2-tan-10.1177_17562864251410787.jpeg]

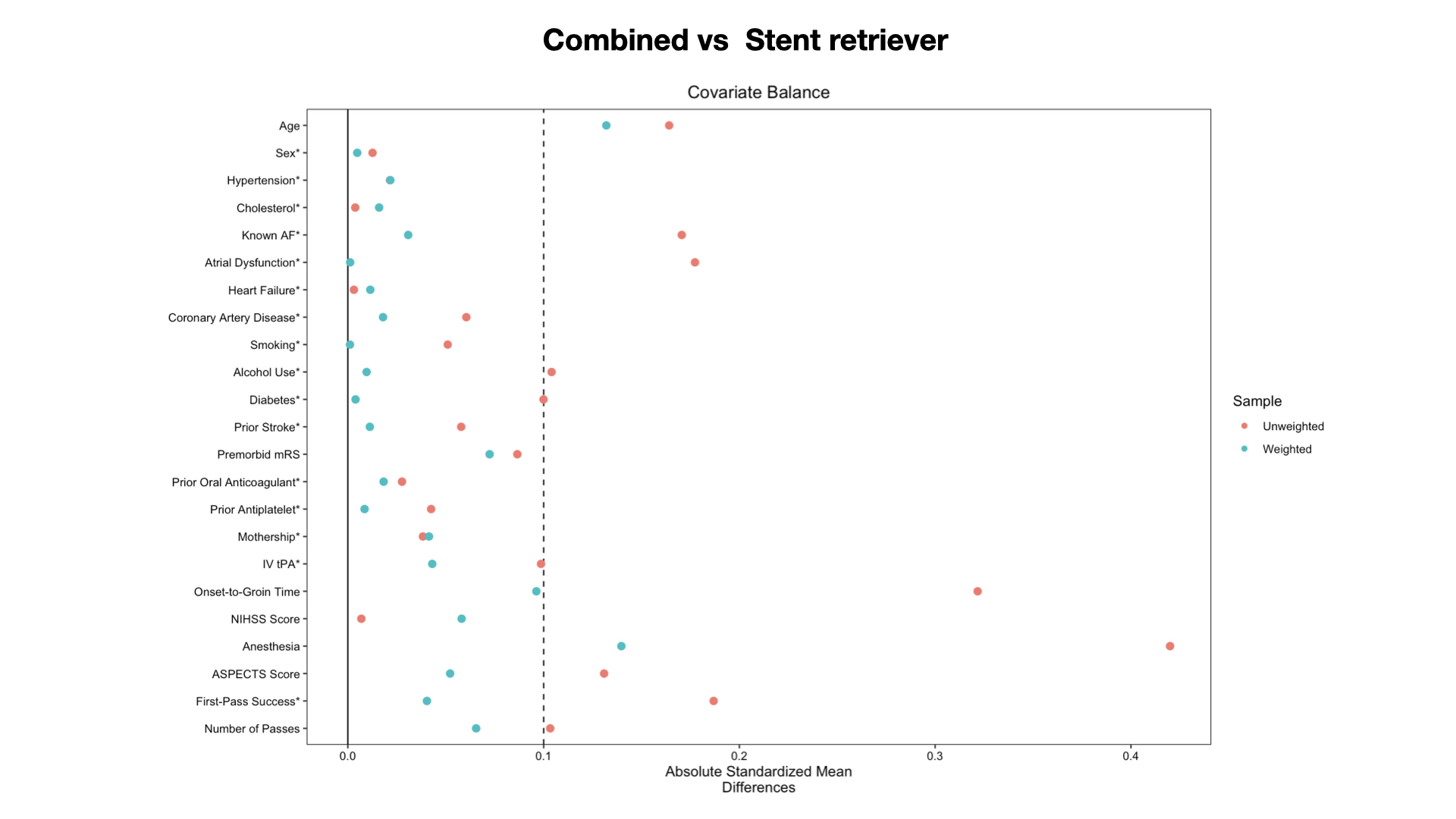

Supplement: sj-jpeg-3-tan-10.1177_17562864251410787 – Supplemental material for Aspiration, stent retriever, or combined approach for basilar artery occlusion: a three-way comparative analysis [file sj-jpeg-3-tan-10.1177_17562864251410787.jpeg]
